# Telemedicine Visits in US Skilled Nursing Facilities

**DOI:** 10.1001/jamanetworkopen.2023.29895

**Published:** 2023-08-18

**Authors:** Agne Ulyte, Ateev Mehrotra, Andrew D. Wilcock, Gillian K. SteelFisher, David C. Grabowski, Michael L. Barnett

**Affiliations:** 1 Department of Health Care Policy, Harvard Medical School, Boston, Massachusetts; 2 Division of General Medicine, Beth Israel Deaconess Medical Center, Boston, Massachusetts; 3 Department of Health Policy and Management, Harvard T.H. Chan School of Public Health, Boston, Massachusetts; 4 Division of General Internal Medicine and Primary Care, Department of Medicine, Brigham and Women’s Hospital, Boston, Massachusetts

## Abstract

**Importance:**

Telemedicine in skilled nursing facilities (SNFs) has the potential to improve access and timeliness of care. During the COVID-19 pandemic in 2020 to 2022, telemedicine coverage expanded, but little is known about patterns of use in SNFs.

**Objective:**

To describe patterns of telemedicine use in SNFs.

**Design, Setting, and Participants:**

This cohort study used 2018 to 2022 Medicare fee-for-service claims and Minimum Data Set 3.0 records to identify short- and long-term care SNF residents. Clinician visits were grouped into routine SNF visits (ie, regular primary care within SNF) and other outpatient visits (ie, with non-SNF affiliated primary and specialty care clinicians). Using a difference-in-differences approach, assessments included whether off-hours visits (measured as weekend visits), new specialist visits, psychiatrist visits, or visits for residents with limited mobility changed differentially between 2018 to 2019 and 2020 to 2021 for SNFs with high compared with low telemedicine use in 2020.

**Exposure:**

Telemedicine adoption at SNF after 2020.

**Main Outcomes and Measures:**

Number and proportion of telemedicine SNF and outpatient visits.

**Results:**

Across 15 434 SNFs and 4 463 591 residents from the period January 2019 through June 2022 (mean [SD] age, 79.7 [11.6] years; 61% female in 2020), telemedicine visits increased from 0.15% in January 2019 to February 2020 to 15% SNF visits and 25% outpatient visits in May 2020. By 2022, telemedicine dropped to 2% of SNF visits and 8% of outpatient visits. The proportion of SNFs with any telemedicine visits annually dropped from 91% in 2020 to 61% in 2022. The facilities with high telemedicine use were more likely to be rural (adjusted odds ratio vs urban, 2.06; 95% CI, 1.77 to 2.40). Psychiatry visits differentially increased in high vs low telemedicine-use SNFs (20.2% relative increase; 95% CI, 1.2% to 39.2%). In contrast, there was little change in outpatient visits for residents with limited mobility (7.2%; 95% CI, −0.1% to 14.6%) or new specialist visits (−0.7%; 95% CI, −2.5% to 1.2%).

**Conclusions and Relevance:**

In this cohort study of SNF residents, telemedicine was rapidly adopted in early 2020 but subsequently stabilized at a low use rate that was nonetheless higher than before 2020. Higher telemedicine use in SNFs was associated with improved access to psychiatry visits in SNFs. A policy to encourage continued telemedicine use may facilitate further access to important services as the technology matures.

## Key Points

**Question:**

How was telemedicine adopted in US skilled nursing facilities (SNFs) during the COVID-19 pandemic in 2020 to 2022?

**Findings:**

In this cohort study of more than 4.4 million residents at 15 434 SNFs, telemedicine visits increased from 0.15% to 15% of routine SNF visits and 37% of other outpatient visits in SNFs in early 2020, before dropping again and stabilizing at 2% of routine SNF and 10% of outpatient visits by mid-2021. Higher telemedicine use was associated with improved access to psychiatry visits in SNFs.

**Meaning:**

In this study, after transiently high use in 2020, telemedicine remained present in SNFs at lower levels by 2022, with higher use associated with more frequent psychiatry visits.

## Teaser

This cohort study examines Medicare data for residents in skilled nursing facilities to determine patterns in the use of telemedicine during the COVID-19 pandemic.

## Introduction

Telemedicine has long been regarded as a promising mechanism to improve access to health care in skilled nursing facilities (SNFs).^1,2,3,4,5^ Clinicians are rarely on site at SNFs in the evening or over the weekend.^6,7,8,9^ Medical issues that present during these off hours often result in unnecessary visits to the emergency department.^10^ Further, SNF residents typically have to leave their facility to access specialty care, making access more challenging for residents with limited mobility. The resulting delays or absence of care contributes to avoidable hospitalizations and emergency care visits.^8,10,11,12^

Despite recognition of its potential,^3^ telemedicine use in SNFs was rare before 2020, and in the Medicare program, it was only reimbursable in rural communities for select types of visits.^4^ This changed when Medicare expanded coverage at the start of the COVID-19 pandemic in early 2020. Telemedicine was allowed for any evaluation and management (E&M) visit for SNF residents, with the goals of limiting the spread of COVID-19 and safely expanding access to needed care.^4,13,14^

Little is known about the potential effect of greater telemedicine use in SNFs,^15,16,17,18^ including whether it could potentially alleviate long-standing gaps in access to specialists or urgent care after hours. As policymakers debate the future of telemedicine reimbursement beyond the COVID-19 emergency, understanding patterns of adoption can guide policy and regulations to help telemedicine improve access to care in SNFs.

We examined trends in telemedicine visits for Medicare SNF residents from January 2019 through June 2022. We profiled the characteristics of telemedicine visits in 2020 to 2021, including the SNFs, clinicians, and patients using it, and examined whether higher adoption of telemedicine was associated with relatively improved access to specialists, care over the weekend, and visits for residents with limited mobility.

## Methods

The study was approved by the Office of Human Research Administration at Harvard T. H. Chan School of Public Health. The requirement for informed consent was waived because the data were deidentified. We followed the Strengthening the Reporting of Observational Studies in Epidemiology (STROBE) reporting guideline for cohort studies.

### Data Sources

We used 100% fee-for-service Medicare administrative claims and the Minimum Data Set version 3.0 files from 2018 to 2022 to identify short- and long-term SNF residents and their E&M visits and Master Beneficiary Summary files for resident characteristics. We added SNF characteristics from the Centers for Medicare & Medicaid Services Nursing Home Compare^19^ data set (January 2020).

### Study Population

We identified postacute (short-term) SNF stays with Medicare Part A SNF claims and long-term care stays using dates obtained from Minimum Data Set assessment records from 2018 to 2022 (through June). Based on a validated algorithm,^20,21^ beneficiaries were considered long-term care residents from the first SNF stay of which any portion was not covered by a Medicare Part A SNF claim (ie, recorded only in the Minimum Data Set). We excluded beneficiaries who did not have full coverage with traditional Medicare Part A or Part B at any point during their SNF stay. We also excluded SNF stays outside the 50 US states and Washington, DC (attrition is shown in eTable 1 in Supplement 1).

### Measuring SNF and Outpatient Visits

The study focused on E&M visits provided in person or via telemedicine for beneficiaries during SNF stays. We identified visits in the Carrier and Outpatient files using the Healthcare Common Procedure Coding System (HCPCS) codes defined as E&M care under the Restructured BETOS Classification System.^22^ Evaluation-and-management critical care, hospital inpatient, observation care, and emergency department services were not included, and we limited services to those provided by prescribing, patient-facing specialists, such as physicians, nurse practitioners, and physician assistants (included specialties are listed in eAppendix 1 in Supplement 1).

We grouped the E&M visits into 2 mutually exclusive groups: (1) routine SNF visits (BETOS subcategory of E&M visits for nursing home services) and (2) other outpatient visits (all other included visits). SNF visits are federally required at regular intervals for all residents in SNFs serving Medicare patients, almost always happen at the facility, and are often delivered by SNF-affiliated clinicians. Apart from such SNF visits, residents can attend outpatient visits with primary or specialty care clinicians, which usually happen outside the facility and often with an established care clinician from before transfer to the SNF.^23^ Because these 2 visit groups are distinct in their clinical purpose, regulations, and reimbursement, we analyzed them separately.

### Study Outcomes

We identified telemedicine visits based on the place of service, HCPCS, or modifier codes recorded on claims (eTable 2 in Supplement 1). We extracted the primary diagnosis of each visit and grouped them into clinical domains.^24^ Multiple SNF (or outpatient) visits by the same clinician for the same patient on a given day were considered just 1 SNF (or outpatient) visit. In the few cases of both telemedicine and in-person services recorded on the same day (eg, telemedicine visit in the Carrier file and in-person visit in the Outpatient file), the visit was considered telemedicine.

We hypothesized that telemedicine could effectively increase the number of accessible clinicians (especially during off hours), facilitate visits where physical examination is less necessary, and reduce barriers associated with transportation and initiating a new specialist relationship. To test these hypotheses, we examined 4 outcomes: (1) SNF visits on weekends; (2) outpatient visits for patients with limited mobility; (3) new specialist physician visits (identified with HCPCS codes 99201-5); and (4) outpatient and geriatric psychiatrist visits. Because the time of a visit is not captured in claims data, we used weekend dates to capture off-hours care.

### Study Variables

We captured beneficiary characteristics, such as sex, age, race and ethnicity, reason for Medicare enrollment, dual Medicaid and Medicare enrollment, and comorbidities (identified with Chronic Conditions Data Warehouse flags)^25^ and flagged beneficiaries who had any outpatient COVID-19 principal diagnosis (code U07.1) during their SNF stay. Race and ethnicity were self-reported at the time of Medicare enrollment and were included to identify potential disparities in telemedicine use. Resident mobility was considered limited for residents with total dependence for locomotion as recorded in the first valid Minimum Data Set assessment in the current or preceding year.

We extracted clinician gender, year of graduation, and zip code from the Centers for Medicare & Medicaid Services Provider Data Catalog.^26^ We characterized SNFs by the proportion of their residents in 2020 who were dually eligible or of a specific race and ethnicity, urban or rural location,^27^ ownership type (for-profit, not-for-profit, government), chain status, Nursing Home Compare^19^ star rating, and staffing level (sum of registered nurse and licensed practical nurse hours per resident-day).

Based on highly concentrated telemedicine use for SNF visits across clinicians, we defined high users as clinicians in the top decile by telemedicine. Following a slightly less skewed distribution across SNFs, we defined high telemedicine-use SNFs as the top quartile of facilities by the proportion of telemedicine visits and low telemedicine-use SNFs as those in the bottom quartile. We used the start and end dates of SNF stays to calculate the number of SNF resident-days overall and during weekends, in order to account for differences in length of residence and express the outcomes per resident-year (365 days) or resident-month (30 days).

### Statistical Analysis

We compared the characteristics of patients, SNFs, and clinicians with high vs lower telemedicine use in 2020 to 2021. Given a highly skewed distribution of telemedicine use among SNFs and clinicians, we modeled the binary outcome of whether or not an SNF or clinician was a high telemedicine user with logistic regressions. We used stratified logistic regression to quantify resident characteristics associated with any telemedicine use within an SNF, with an offset (natural logarithm of SNF stay duration in days) to account for the higher probability of any visit (including telemedicine) during a longer stay. Given the changing nature of the COVID-19 pandemic, in a sensitivity analysis, we compared SNF characteristics predictive of high telemedicine use separately for 2020 and 2021. Data analysis was done with SAS version 7.15 (SAS Institute). Regression models with SNF fixed effects were run with Stata version 17 (StataCorp). Differences were considered statistically significant with 2-sided *P* ≤ .05.

We tested whether higher uptake of telemedicine in SNFs was associated with improved access to care in 2020 to 2021 in 4 scenarios. Using a difference-in-differences approach, we compared high telemedicine-use SNFs in 2020 (for SNF or outpatient visits, depending on the studied outcome) to low-use SNFs. We included only residents in long-term care with at least 60 SNF days in a calendar year, because patients staying at SNFs long-term and receiving their main care there would be expected to be affected by changing care patterns within an SNF the most. We included only SNFs in continuous operation before and during the pandemic, defined as SNFs with 10 or more SNF visits and 10 or more outpatient visits in 2018-2019 and 2020-2021.

We modeled the number of visits per resident within a calendar year using linear regression adjusting for patient characteristics, year, the state where the SNF is located, SNF telemedicine-use category (high or low), and an interaction term between this category and period (2018-2019 or 2020-2021), which captures the difference-in-differences. We weighted the modeled patient-level observations by the numbers of days the resident spent in that SNF that year and clustered model errors by SNF. Parallel trends in the outcomes in high and low telemedicine-use SNFs were tested in 2018 to 2019. We compared the outcomes in 2020 and 2021 separately in a model with 2 interaction terms.

## Results

### Overall Patterns of Telemedicine Use

From January 2019 through June 2022, 2 761 128 short- and 2 147 944 long-term care residents (4 463 591 unique beneficiaries) stayed in 15 434 SNFs. In 2020, the mean (SD) age of short-term residents was 79.4 (10.7) years (80.0 [12.2] years for long-term residents; 79.7 [11.6] years overall), 59% were women (63% of long-term residents; 61% overall), and 9.8% were Black (13.4% of long-term residents; 12% overall) (eTable 3 in Supplement 1).

Telemedicine visits were concentrated within highest-using SNFs: 50% of telemedicine SNF visits in 2020 to 2021 were provided by the top 18% of telemedicine-using SNFs and 80% of visits by the top 39%. Telemedicine use was even more concentrated among clinicians: 50% of telemedicine SNF visits were provided by the top 7% of telemedicine-using clinicians, and 80% of visits by the top 13%.

### Telemedicine Trends in 2019 to 2022

**Figure 1.**
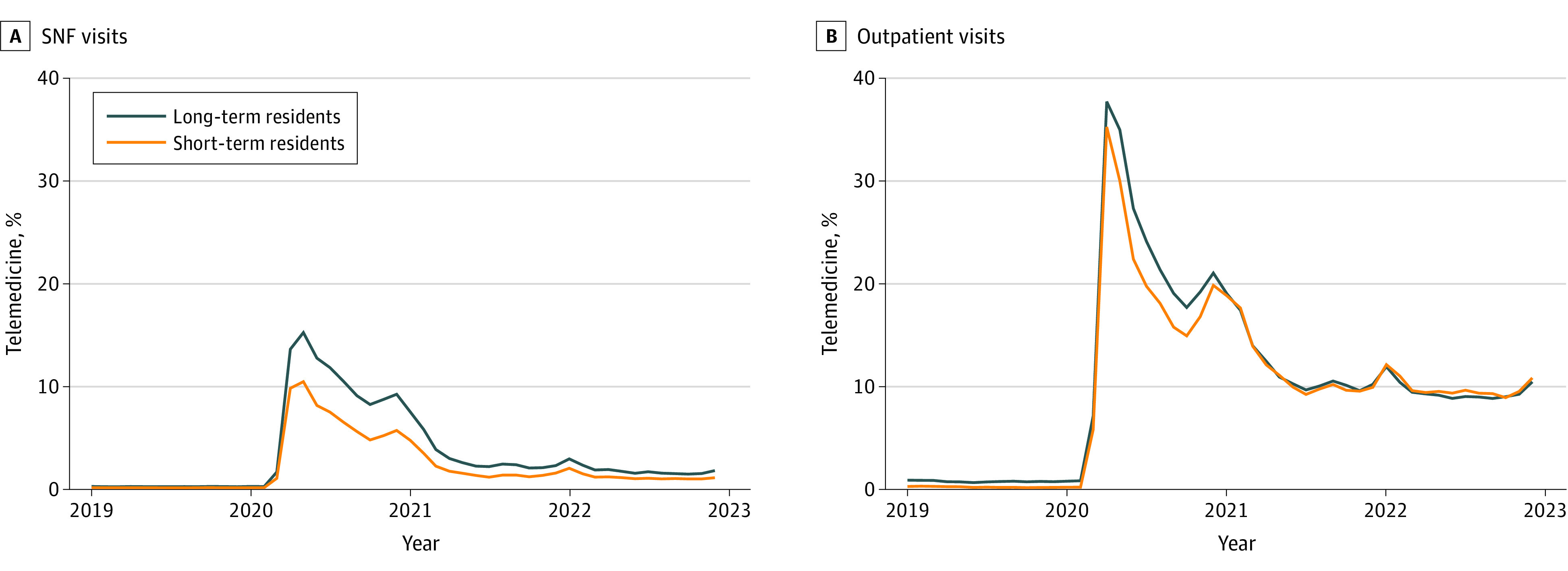
Proportion of Skilled Nursing Facility (SNF) and Outpatient Visits Delivered by Telemedicine for Short- and Long-Term SNF Residents in 2019 to 2022 The group SNF visits (A) captures regular primary care encounters within SNFs, and the outpatient visits group (B) captures visits with primary and specialty care clinicians who are not affiliated with SNFs.

Before 2020, telemedicine visits constituted 0.15% of all visits for SNF residents (Figure 1). After an initial increase in early 2020 to 15% of SNF visits and 37% of outpatient visits, the proportion of subsequent telemedicine visits decreased gradually, reaching a plateau of 2% for SNF visits and 10% for outpatient visits in summer 2021 and 2% for SNF visits and 8% for outpatient visits by 2022. Among all SNFs, 823 SNFs (5%) used telemedicine at least once in 2019, 13 920 (91%) in 2020, 12 383 (81%) in 2021, and 9321 (61%) in the first half of 2022.

**Figure 2.**
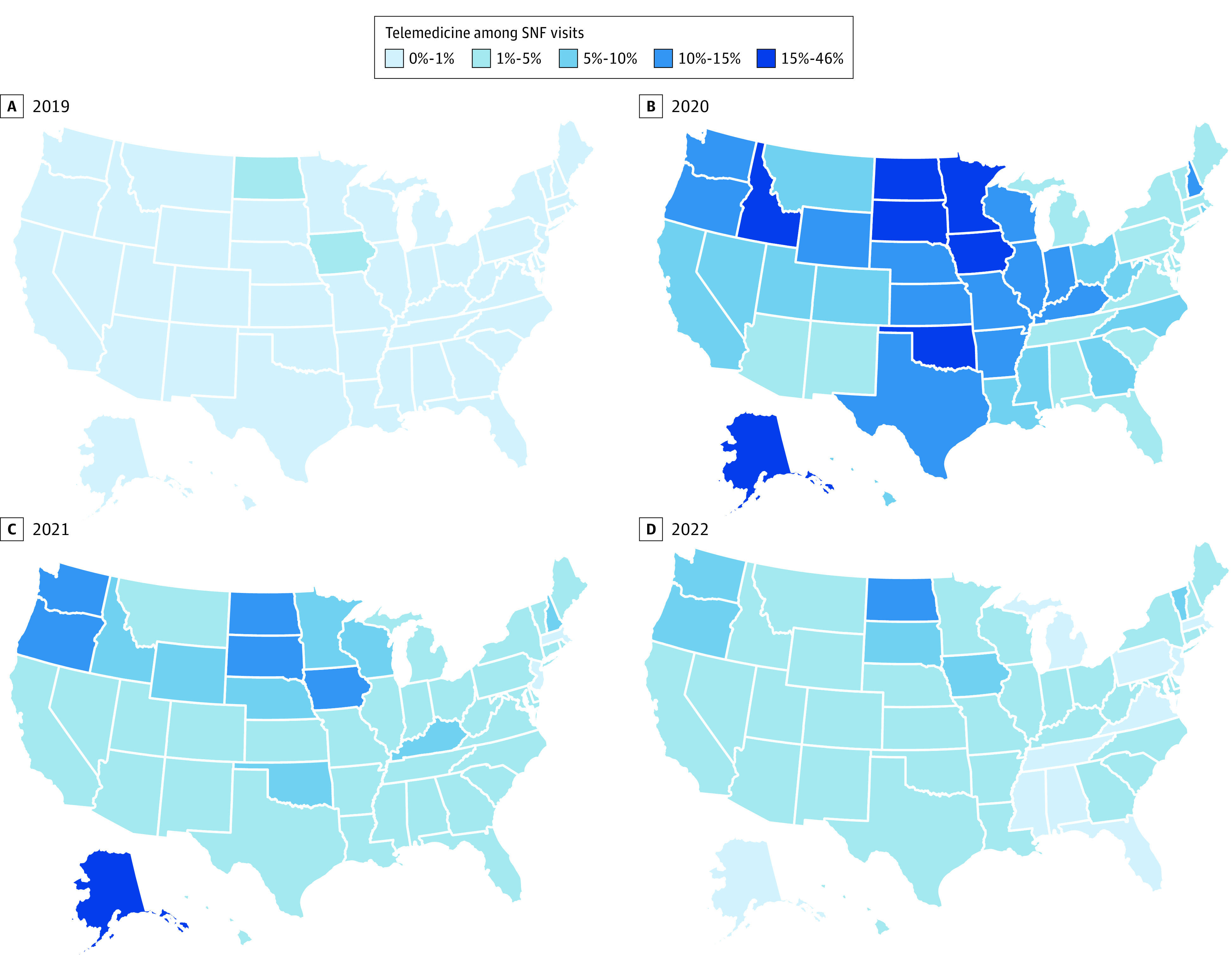
Telemedicine Use for Skilled Nursing Facility (SNF) Visits Across US States in 2019 to 2022 The map for 2022 (D) depicts visits in January through June 2022.

In 2019, only 3 Midwestern states had more than 1% of SNF visits delivered via telemedicine (Figure 2 and eFigure 1 in Supplement 1 shows outpatient visits). In 2020, all states used telemedicine for more than 1% and 20 states for more than 10% of SNF visits. By the first half of 2022, only 1 state used telemedicine for more than 10% of SNF visits and 8 states returned to less than 1% of use.

### Patterns of Use for Short- and Long-Term Care Residents

Although the overall trend of telemedicine use was similar for short- and long-term care residents, the proportion of telemedicine visits was slightly higher for long-term care residents (Figure 1). Short-term care residents had a higher absolute rate of telemedicine visits due to overall more frequent visits during their temporary stays (eFigure 2 in Supplement 1). COVID-19 was the most frequent diagnosis for telemedicine visits among short-term residents (6.4% of all telemedicine visits) (eTable 4 in Supplement 1). Mental health diagnoses were the most common among telemedicine visits of long-term care residents (depressive disorders, 8.2%, and neurocognitive disorders, 7.9% of all visits).

### Characteristics of SNFs, Clinicians, and Patients With Telemedicine Use in 2020 to 2021

The top quartile (3609) of SNFs used telemedicine for at least 9.8% (median 18.4% in top quartile vs median 1.7% in other SNFs). High-use SNFs were more likely to be situated in the Midwest or West (adjusted odds ratio [aOR], 1.44; 95% CI, 1.30-1.58, and aOR, 1.24; 95% CI, 1.08-1.42, respectively, compared with the South), less likely to be in the Northeast (aOR, 0.39; 95% CI, 0.33-0.45), and more likely to be in nonmetropolitan areas (aOR, 2.06; 95% CI, 1.77-2.40, rural vs metropolitan) (Table 1).^27^ These SNFs were also smaller (aOR, 0.68; 95% CI, 0.61-0.76, for ≥121 beds vs ≤80 beds) and served fewer Black residents (8.7% vs 12.7%). Adjusted odds ratios were similar when studying high-use SNFs in 2020 and 2021 separately, except for SNF location (eTable 5 in Supplement 1). High telemedicine-use clinicians practiced in more rural areas and served more SNFs (mean 15 vs 10; eTable 6 in Supplement 1).

**Table 1.** Characteristics of SNFs in the Top Quartile by Telemedicine Use for SNF Visits in 2020 to 2021^a^

	SNFs by telemedicine use, No. (%)		Adjusted odds ratio (95% CI)
	High	Lower	
No. of SNFs	3609	10 842	NA
No. of telemedicine visits in the group	1 687 008	989 088	NA
Proportion of telemedicine visits, median (IQR), %	18.4 (13.2-29.3)	1.7 (0.4-4.2)	NA
Ownership			
For profit	2573 (71.3)	7667 (70.7)	1 [Reference]
Government	244 (6.8)	599 (5.5)	0.83 (0.69-0.99)
Not for profit	792 (22.0)	2576 (23.8)	0.95 (0.85-1.05)
Part of chain			
Yes	2527 (70.0)	7739 (71.4)	0.93 (0.85-1.03)
No	1082 (30.0)	3103 (28.6)	1 [Reference]
Region			
South	1173 (32.5)	3924 (36.2)	1 [Reference]
Midwest	1606 (44.5)	3121 (28.8)	1.44 (1.30-1.58)
Northeast	242 (6.7)	2214 (20.4)	0.39 (0.33-0.45)
West	588 (16.3)	1583 (14.6)	1.24 (1.08-1.42)
Geography^b^			
Metropolitan	1954 (54.1)	8052 (74.3)	1 [Reference]
Micropolitan	725 (20.1)	1276 (11.8)	1.86 (1.66-2.07)
Small urban	519 (14.4)	968 (8.9)	1.59 (1.40-1.80)
Rural	411 (11.4)	546 (5.0)	2.06 (1.77-2.40)
No. of certified beds			
≤80	1531 (42.4)	3372 (31.1)	1 [Reference]
81-120	1328 (36.8)	3958 (36.5)	0.81 (0.74-0.90)
≥121	750 (20.8)	3512 (32.4)	0.68 (0.61-0.76)
Overall star rating^c^			
1-2	1429 (39.6)	3910 (36.1)	1 [Reference]
3-4	1416 (39.2)	4332 (40.0)	0.87 (0.79-0.95)
5	764 (21.2)	2600 (24.0)	0.83 (0.74-0.94)
Nurse hours per resident-day			
<1.3	1200 (33.3)	2843 (26.2)	1 [Reference]
1.3-1.7	1487 (41.2)	4695 (43.3)	0.82 (0.75-0.91)
>1.7	922 (25.6)	3304 (30.5)	0.74 (0.66-0.83)
Residents with Medicaid^d^			
≤50%	1126 (31.2)	3974 (36.7)	1 [Reference]
51%-70%	1098 (30.4)	3260 (30.1)	1.11 (1.00-1.24)
≥71%	1385 (38.4)	3608 (33.3)	1.53 (1.36-1.71)
Resident race and ethnicity, mean (SD), %^e^			
Black	8.7 (15.0)	12.7 (17.3)	0.87 (0.84-0.89)
White	86.1 (17.7)	81.9 (19.8)	1 [Reference]
Other	5.3 (9.3)	5.4 (9.5)	0.99 (0.95-1.04)

Abbreviations: NA, not applicable; SNFs, skilled nursing facilities.

^a^ SNFs in the high telemedicine-use group had more than 9.8% of their SNF visits delivered in telemedicine. Included are SNFs that had at least 10 SNF visits for their residents in both 2020 and 2021. Ownership, chain, bed, and rating information is from January 2020 (Centers for Medicare & Medicaid Services Nursing Homes Compare). SNFs with missing information in Centers for Medicare & Medicaid Services Nursing Homes Compare are excluded (n = 800).

^b^ Area type is classified based on Rural-Urban Commuting Area Codes^27^: metropolitan (1-3), micropolitan (4-6), small urban (7-9), or rural area (10).

^c^ Medicare star ratings range from 1 (much below average) to 5 (much above average). This score is a composite ranking of individual SNFs that incorporates multiple measures of SNF quality, staffing, and health inspection performance.

^d^ The proportion of Medicaid-covered residents among all Medicare residents within the SNF.

^e^ The mean proportion of residents with a specific race and ethnicity represents the proportion of unique Medicare beneficiaries residing in the SNFs of that group. Odds ratios are provided for the variable scaled by 10 (the odds of an SNF being in a high telemedicine-use group associated with a 10 percentage point increase in the race and ethnicity among all Medicare residents). The other race and ethnicity group includes the categories Asian, Hispanic, North American Native, other, and missing.

Among SNF residents, 808 530 (25.5%) received at least 1 SNF or outpatient telemedicine visit during their stay (eTable 7 in Supplement 1). Adjusting for length of stay, residents younger than 65 years (vs 65-74 years; aOR, 1.26; 95% CI, 1.24-1.28) and with a COVID-19 diagnosis during their stay (aOR, 1.69; 95% CI, 1.67-1.70) were more likely to receive telemedicine visits. Residents in long-term care (aOR, 0.55; 95% CI, 0.55-0.55) as well as older, non-White, dual-eligible residents were less likely to receive telemedicine visits.

### Change in Access to Care in High Telemedicine-Adopting SNFs

**Figure 3.**
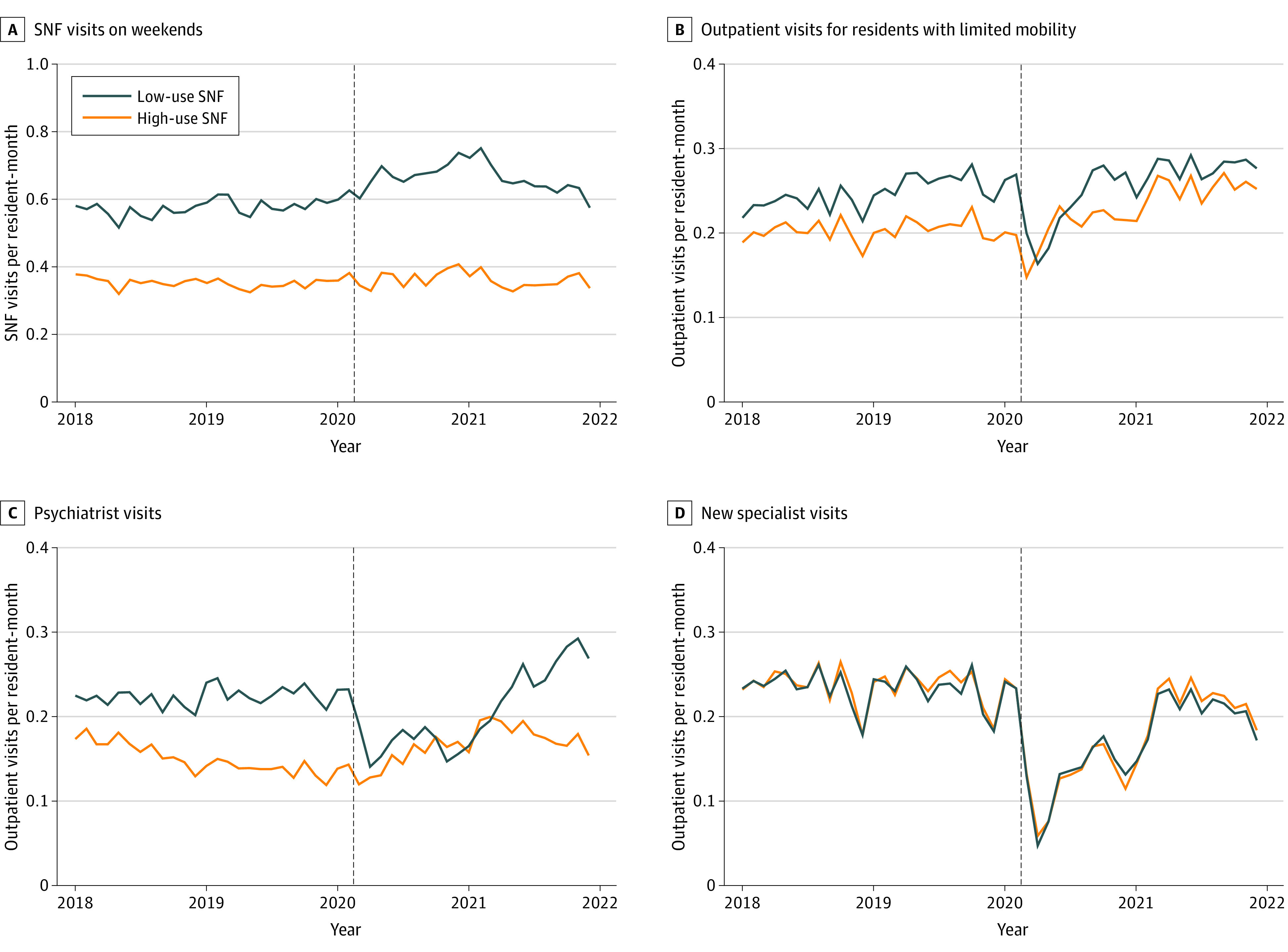
Number of Visits per Resident-Month in 2018 to 2021 in Skilled Nursing Facilities (SNFs) With High or Low Telemedicine Use High-use SNFs were defined as those in the top quartile by telemedicine use in 2020 for SNF or outpatient visits (depending on the analyzed outcome) and low-use SNFs as those in the bottom quartile.

We examined the outcomes of high telemedicine adoption in 4 scenarios, corresponding to situations where telemedicine could be expected to increase access to care. Figure 3 shows the unadjusted change in total visits in these scenarios in high vs low telemedicine-use SNFs (the proportion of telemedicine shown in eFigure 3 in Supplement 1). Pre-trends were parallel in high- and low-use SNFs for new specialist visits but not the other outcomes (eAppendix 2 in Supplement 1). Compared with 2018 to 2019, high-use SNFs in 2020 to 2021 provided more psychiatry visits per resident year than other SNFs (0.03; 95% CI, 0.00 to 0.07), a 20.2% relative increase (95% CI, 1.2% to 39.2%) compared with the 2019 mean in high-use SNFs (Table 2 and model details in eAppendix 3 in Supplement 1), with higher relative increase in 2020 (eAppendix 4 in Supplement 1). High-use SNFs also provided more outpatient visits for residents with limited mobility (adjusted difference, 0.18; 95% CI, 0.00 to 0.37), a 7.2% relative increase (95% CI, −0.1% to 14.6%), with a higher increase in 2021. High-use SNFs had fewer SNF visits on weekends (relative change of −20.1%; 95% CI, −29.3% to −11.0%) and no difference in new outpatient visits with specialist physicians (relative change of −0.7%; 95% CI, −2.5% to 1.2%).

**Table 2.** Difference-in-Differences Between the Change in Visits in 2018-2019 and 2020-2021 in High vs Low Telemedicine-Use SNFs

Patient group: visits per resident-year	Visits per resident-year in 2019 in high-use SNFs	Difference-in-differences		
		Estimate (95% CI)	Relative change, % (95% CI)	*P* value
Residents on weekends: SNF visits on weekends	4.22	−0.85 (−1.24 to −0.46)	−20.1 (−29.3 to −11.0)	<.001
Residents with limited mobility: outpatient visits	2.53	0.18 (0.00 to 0.37)	7.2 (−0.1 to 14.6)	.05
All residents: outpatient psychiatrist visits	0.17	0.03 (0.00 to 0.07)	20.2 (1.2 to 39.2)	.04
All residents: new outpatient specialist visits	0.29	0.00 (−0.01 to 0.00)	−0.7 (−2.5 to 1.2)	.49

Abbreviation: SNFs, skilled nursing facilities.

## Discussion

To our knowledge, this study offers the first comprehensive picture of telemedicine adoption for SNF residents after policy changes in the COVID-19 pandemic, when telemedicine was rapidly implemented to prevent infection spread during in-person encounters.^28^ We found that telemedicine use increased rapidly in early 2020, driven by the highest-using SNFs and clinicians. However, by mid-2021, the share of telemedicine decreased to 2% among SNF and 10% among outpatient visits, consistent with similar trajectories in other settings.^29,30^ Overall, telemedicine use did not result in a substantially different volume of visits, mitigating concerns that loosening restrictions and regulations might unleash potential abuse and excessive billing. Contrary to previous concerns about the barrier of the start-up investment to enable telemedicine,^1^ rapid expansion to the majority of SNFs in 2020 demonstrates that telemedicine could be ramped up without a long and intensive set-up process.

High telemedicine use had a mixed association with changes in care delivery for the 4 scenarios we considered. We found evidence that higher adoption of telemedicine was associated with improved access to psychiatry visits and outpatient care for residents with low mobility. While low-telemedicine SNFs had a large decrease in psychiatry visits in 2020, the level remained steady in high-telemedicine SNFs. Likely, telemedicine helped maintain established patient-clinician connections and expanded access to potential new clinicians, especially amid the long-standing decline in psychiatrist numbers.^31^ Despite the potential to improve off-hours care with telemedicine,^32^ SNF visits on weekends unexpectedly increased more in low-use SNFs, although nonparallel pre-trends limit the interpretation of this finding. In addition, by 2021 overall telemedicine use for new specialist visits and SNF visits on weekends was relatively low. Additional use cases might still become more prevalent with time if telemedicine becomes a more integral part of care at SNFs.

Taken together, these results suggest that though telemedicine could provide an opportunity to extend the temporal and physical boundaries of care in SNFs, in practice this did not happen consistently.^2,18,23^ One exception may be in rural areas with a shrinking physician workforce, where telemedicine might be the only feasible option for receiving timely care.^18,33^ We found that clinicians using the most telemedicine were more likely to serve rural areas and visited more SNFs. Access to mental health care was also particularly important as depressive symptoms increased in nursing home residents during the pandemic, likely as a result of restricted visiting and increased isolation.^34,35^

Telemedicine use was also highly concentrated among a small group of clinicians, suggesting that staff and clinician preferences were likely an important driver in the magnitude of telemedicine implementation. In a small qualitative study, SNF staff deemed that telemedicine visits of routine care were inferior to a physician actually visiting the facility and noted that telemedicine encounters increased staff workload because of new and redundant tasks that were not offset with reduction in other responsibilities.^18^ In interviews, physicians practicing at SNFs also noted that while organization resources are critical, staff time to prepare for and facilitate a telemedicine encounter is an even larger bottleneck.^36^ Increased workload combined with staffing shortages, preferences of the older patients typically residing in nursing homes,^37,38^ and a lack of investment in technology^18,39^ could have led to telemedicine being used only transiently in 2020 to 2021.

### Limitations

Our study has limitations. First, we report telemedicine use only in Medicare fee-for-service beneficiaries, so these results may apply less to residents with different coverage policies, such as Medicare Advantage or Medicaid. Second, we relied on a broad set of indicators in Medicare claims to define telemedicine visits. However, these indicators do not capture the diversity of visit formats, ranging from audio-only calls to technology-facilitated remote physical examinations. We also could not capture other remote services, such as phone consultations directly between a physician and nurse. Third, we are not able to draw causal conclusions of how telemedicine influenced residents’ care and outcomes, as its adoption was most likely nonrandom, particularly in scenarios with existing pre-trends.

## Conclusions

This study found that although virtually all policy barriers to telemedicine use in SNFs were removed at the start of the pandemic in 2020, its use remained concentrated in a small proportion of SNFs, especially in 2021 to 2022. We found preliminary evidence that telemedicine adoption might be associated with some changes in patterns of clinical care, potentially leading to improved access to specialty care. Continued reimbursement of telemedicine services in SNFs thus has a potential to improve resident care without substantially increasing its overall volume.

## Notes

Supplement 1. **eTable 1.** Attrition of SNFs and their residents included in the study **eAppendix 1.** List of included clinician specialties **eTable 2.** List of place-of-service, procedure, and modifier codes to identify telemedicine visits **eTable 3.** Characteristics of long- and short-term care residents in SNFs in 2020 **eFigure 1.** Telemedicine use for outpatient visits in SNF residents across US states in 2019-2022 **eFigure 2.** Count of SNF and outpatient visits delivered by telemedicine for short- and long-term SNF residents in 2019-2022 **eTable 4.** Top principal diagnoses groups in telemedicine visits in 2020-2021 **eTable 5.** Adjusted odds ratios of characteristics of SNFs in the top quartile by telemedicine use for SNF visits in 2020 and 2021 **eTable 6.** Characteristics of SNF visit providing clinicians in the top decile by telemedicine use in 2020-2021 **eTable 7.** Characteristics of patients who received at least one telemedicine visit during their SNF stay in 2020-2021 **eFigure 3.** Proportion of visits delivered by telemedicine in four examined clinical care scenarios for long-term care residents in high- and low- telemedicine use SNFs in 2018-2021 **eAppendix 2.** Testing of parallel trends for the outcomes before telemedicine expansion **eAppendix 3.** Difference-in-differences in visit counts in 2018-2019 vs 2020-2021 in high vs low telemedicine adopting SNFs **eAppendix 4.** Difference-in-differences models with a separate term for 2020 and 2021

Supplement 2.
Data Sharing Statement
