# Supplementary material for: Telemedicine Visits in US Skilled Nursing Facilities
Source: JAMA Netw Open. 2023 Aug 18;6(8):e2329895. doi: 10.1001/jamanetworkopen.2023.29895 (PMC10439478; doi:10.1001/jamanetworkopen.2023.29895)
Supplement: Supplement 1. — eTable 1. Attrition of SNFs and their residents included in the study eAppendix 1. List of included clinician specialties eTable 2. List of place-of-service, procedure, and modifier codes to identify telemedicine visits eTable 3. Characteristics of long- and short-term care residents in SNFs in 2020 eFigure 1. Telemedicine use for outpatient visits in SNF residents across US states in 2019-2022 eFigure 2. Count of SNF and outpatient visits delivered by telemedicine for short- and long-term SNF residents in 2019-2022 eTable 4. Top principal diagnoses groups in telemedicine visits in 2020-2021 eTable 5. Adjusted odds ratios of characteristics of SNFs in the top quartile by telemedicine use for SNF visits in 2020 and 2021 eTable 6. Characteristics of SNF visit providing clinicians in the top decile by telemedicine use in 2020-2021 eTable 7. Characteristics of patients who received at least one telemedicine visit during their SNF stay in 2020-2021 eFigure 3. Proportion of visits delivered by telemedicine in four examined clinical care scenarios for long-term care residents in high- and low- telemedicine use SNFs in 2018-2021 eAppendix 2. Testing of parallel trends for the outcomes before telemedicine expansion eAppendix 3. Difference-in-differences in visit counts in 2018-2019 vs 2020-2021 in high vs low telemedicine adopting SNFs eAppendix 4. Difference-in-differences models with a separate term for 2020 and 2021 [file jamanetwopen-e2329895-s001.pdf]

Supplemental Online Content

Ulyte A, Mehrotra A, Wilcock AD, SteelFisher GK, Grabowski DC, Barnett ML. Telemedicine visits in US skilled nursing facilities. *JAMA Netw Open*. 2023;6(8):e2329895. doi:10.1001/jamanetworkopen.2023.29895

eTable 1. Attrition of SNFs and their residents included in the study

eAppendix 1. List of included clinician specialties

eTable 2. List of place-of-service, procedure, and modifier codes to identify telemedicine visits

eTable 3. Characteristics of long- and short-term care residents in SNFs in 2020

eFigure 1. Telemedicine use for outpatient visits in SNF residents across US states in 2019-2022

eFigure 2. Count of SNF and outpatient visits delivered by telemedicine for short- and long-term SNF residents in 2019-2022

eTable 4. Top principal diagnoses groups in telemedicine visits in 2020-2021

eTable 5. Adjusted odds ratios of characteristics of SNFs in the top quartile by telemedicine use for SNF visits in 2020 and 2021

eTable 6. Characteristics of SNF visit providing clinicians in the top decile by telemedicine use in 2020-2021

eTable 7. Characteristics of patients who received at least one telemedicine visit during their SNF stay in 2020-2021

eFigure 3. Proportion of visits delivered by telemedicine in four examined clinical care scenarios for long-term care residents in high- and low- telemedicine use SNFs in 2018-2021

eAppendix 2. Testing of parallel trends for the outcomes before telemedicine expansion

eAppendix 3. Difference-in-differences in visit counts in 2018-2019 vs 2020-2021 in high vs low telemedicine adopting SNFs

eAppendix 4. Difference-in-differences models with a separate term for 2020 and 2021

This supplemental material has been provided by the authors to give readers additional information about their work.

© 2023 Ulyte A et al. *JAMA Network Open.*

eTable 1. Attrition of skilled nursing facilities and their residents included in the study

Population	N of residents	N of facilities
Beneficiaries with a SNF stay (Part A claim) or MDS record in 2018-2022 (through June), fully covered by Medicare Part A or Part B during their SNF stay	6,661,788	18,067
Exclude stays in other locations than SNFs (with provider numbers starting not in 5000-6499)	5,991,813	15,794
Exclude if not in one of the 50 US states or DC	5,990,984	15,774
**Difference-in-differences analysis**		
Long-term care residents in 2018-2021, with ≥60 days at a SNF within the calendar year	1,723,131	15,657
Exclude SNFs that had <10 SNF and <10 other resident visits in the periods before (2017-2019) or during (2020-2021) the pandemic	1,715,375	15,061
Exclude residents and SNFs with missing characteristics	1,713,421	14,968

SNF – skilled nursing facility.

© 2023 Ulyte A et al. *JAMA Network Open.*

eAppendix 1. List of included clinician specialties

CMS Provider Specialty code	Specialty
1	General practice
2	General surgery
3	Allergy/immunology
4	Otolaryngology
5	Anesthesiology
6	Cardiology
7	Dermatology
8	Family practice
9	Interventional Pain Management
10	Gastroenterology
11	Internal medicine
12	Osteopathic manipulative therapy
13	Neurology
14	Neurosurgery
16	Obstetrics/gynecology
17	Hospice and Palliative Care
18	Ophthalmology
19	Oral surgery (dentists only)
20	Orthopedic surgery
21	Cardiac Electrophysiology
23	Sports medicine
23	Peripheral vascular disease, medical or surgical (osteopaths only)
24	Plastic and reconstructive surgery
25	Physical medicine and rehabilitation
26	Psychiatry
27	Geriatric Psychiatry
28	Colorectal surgery
29	Pulmonary disease
33	Thoracic surgery
34	Urology
37	Pediatric medicine
38	Geriatric medicine
39	Nephrology
40	Hand surgery
42	Certified nurse midwife
43	CRNA
44	Infectious disease
46	Endocrinology
48	Podiatry
50	Nurse practitioner
66	Rheumatology
72	Pain Management
76	Peripheral vascular disease
77	Vascular surgery
78	Cardiac surgery
79	Addiction medicine
81	Critical care (intensivists)
82	Hematology
83	Hematology/oncology

84	Preventive medicine
85	Maxillofacial surgery
86	Neuropsychiatry
89	Certified clinical nurse specialist
90	Medical oncology
91	Surgical oncology
92	Radiation oncology
93	Emergency medicine
94	Interventional radiology
97	Physician assistant
98	Gynecologist/oncologist
99	Unknown physician specialty
C0	Sleep medicine
C3	Interventional cardiology
C5	Dentist
C6	Hospitalist
C7	Advanced heart failure and transplant cardiology
C8	Medical toxicology
C9	Hematopoietic cell transplantation and cellular therapy

CMS - The Centers for Medicare & Medicaid Services.
The full list of all CMS Provider Specialty codes is accessible at https://resdac.org/sites/datadocumentation.resdac.org/files/CMS_PRVDR_SPCLTY_TB_rev01242018_0.txt

eTable 2. List of place-of-service, procedure, and modifier codes to identify telemedicine visits

Category	Codes
Place of service	01, 02
Healthcare Common Procedure Coding System (HCPCS)	G2025, G0406-8, G2010, G2012, G2061-3, G2250-2, 99421-3, 99441-3, 98970-2, 98966-8
HCPCS modifier codes	GT, GQ, 95, G0, FQ, 93

Any of the codes would suffice to flag a visit as telemedicine. For SNF visits that were selected based on Healthcare Common Procedure Coding System (HCPCS) codes, only place of service and HCPCS modifier codes could be used to identify telemedicine visits.

© 2023 Ulyte A et al. *JAMA Network Open.*

eTable 3. Characteristics of long- and short-term care residents in SNFs in 2020

	Long-term residents	Short-term residents
N	1,078,644 (55.5%)	865,851 (44.5%)
Female sex (%)	674,074 (62.5%)	509,140 (58.8%)
Age (%)		
<65	110,055 (10.2%)	67,348 (7.8%)
65-74	223,086 (20.7%)	205,070 (23.7%)
75-84	298,442 (27.7%)	290,712 (33.6%)
≥85	447,061 (41.5%)	302,721 (35.0%)
Race/ethnicity (%)		
Black	144,505 (13.4%)	84,932 (9.8%)
White	872,205 (80.9%)	734,157 (84.8%)
Other	61,934 (5.7%)	46,762 (5.4%)
Dual eligible in 2019 (%)	795,401 (73.7%)	236,446 (27.3%)
Reason for Medicare enrollment (%)		
Old age	753,200 (69.8%)	667,997 (77.2%)
Disability or renal failure	325,444 (30.2%)	197,854 (22.9%)
N of comorbidities (mean, SD)	11.4 (3.5)	11.5 (3.3)
Dementia (%)	398,013 (36.9%)	137,514 (15.9%)

SNF – skilled nursing facility.
Residents who had both short- and subsequent long-term care stays in 2020 are included in both groups. All residents staying at least one day at a SNF in 2020 are included. Other race/ethnicity includes Asian, Hispanic, North American native, other, and missing.

eFigure 1. Telemedicine use for outpatient visits in SNF residents across US states in 2019-2022

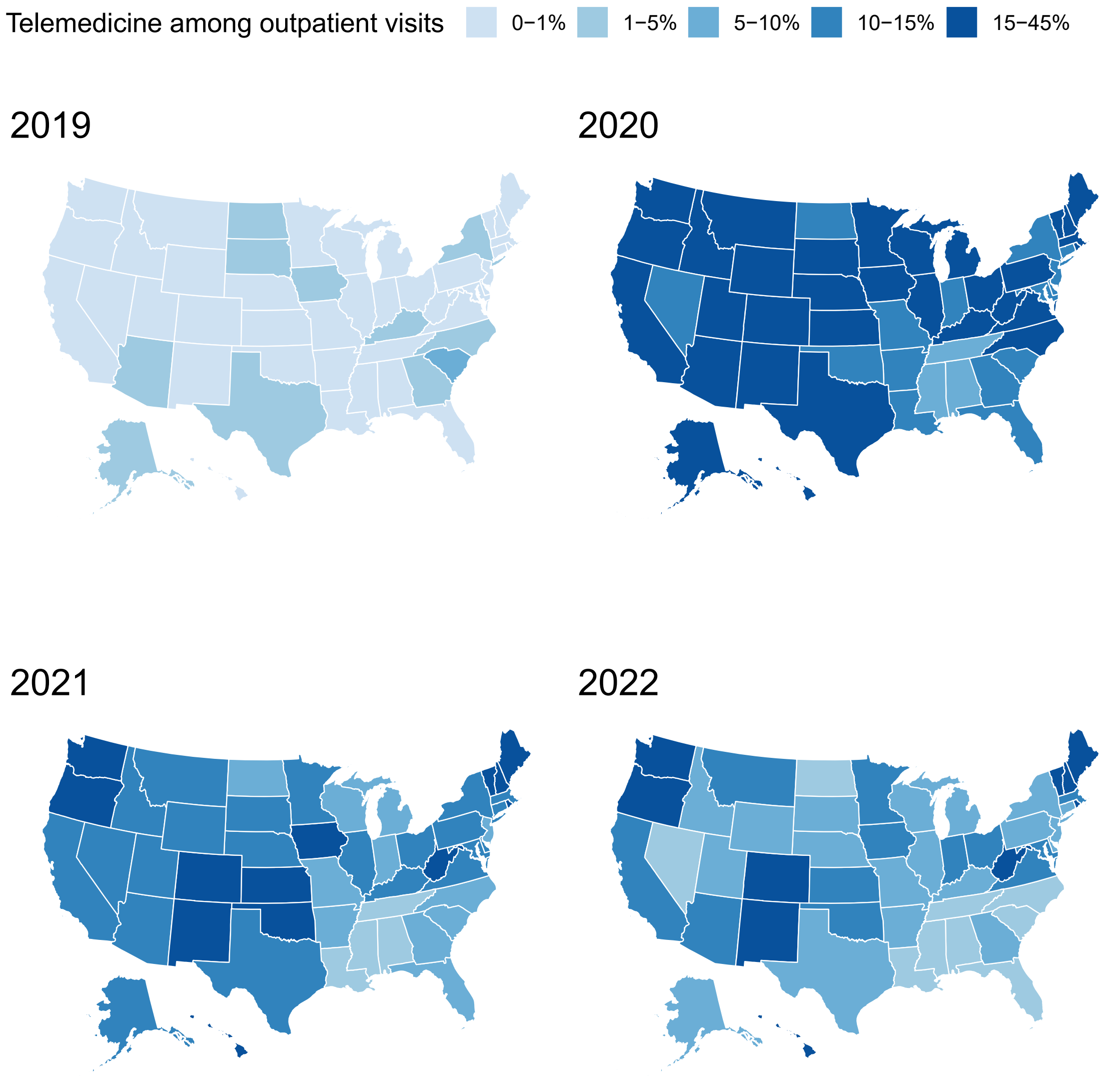

SNF – skilled nursing facility.
Outpatient visits capture visits with non-SNF affiliated primary and specialty care clinicians. Map for 2022 depicts visits in January-June 2022.

© 2023 Ulyte A et al. *JAMA Network Open.*

eFigure 2. Count of SNF and outpatient visits delivered by telemedicine for short- and long-term SNF residents in 2019-2022

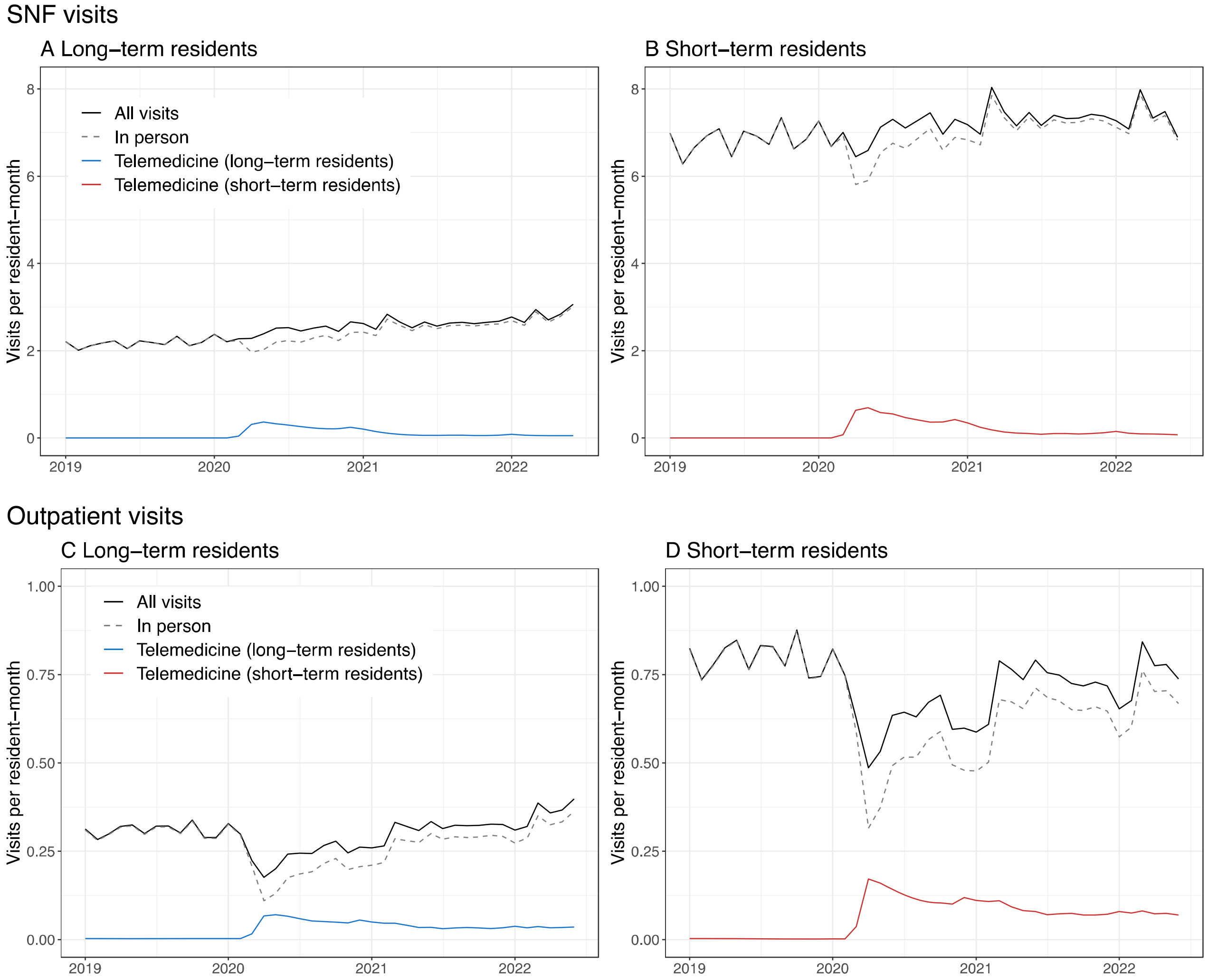

SNF visits are shown in top row (A and B) and outpatient visits in bottom row (C and D). Visits for long-term care residents are on the left (A and C) and visits for short-term stay residents on the right (B and D).

© 2023 Ulyte A et al. *JAMA Network Open.*

eTable 4. Top principal diagnoses groups in telemedicine visits in 2020-2021

	Short-term stays<br>Diagnosis group	TM<br>%	all<br>%	Long-term care<br>Diagnosis group	TM<br>%	all<br>%
1	COVID-19	6.4	2.9	Depressive disorders	8.2	4.6
2	Essential hypertension	4.8	5.7	Neurocognitive disorders	7.9	6.3
3	Malaise and fatigue	3.2	3.7	COVID-19	6.2	2.8
4	Depressive disorders	3.1	1.7	Essential hypertension	6.0	7.0
5	Pressure ulcer of skin	2.8	2.3	Schizophrenia spectrum and other psychotic disorders	4.1	2.3
6	Muscle disorders	2.6	3.5	Pressure ulcer of skin	3.5	3.1
7	Neurocognitive disorders	2.5	1.7	Anxiety and fear-related disorders	3.4	2.2
8	Heart failure	2.5	2.9	Diabetes mellitus with complication	2.4	2.2
9	Nervous system signs and symptoms	2.4	4.0	Other general signs and symptoms	2.0	2.7
10	Other general signs and symptoms	2.2	2.5	Exposure, encounters, screening or contact with infectious disease	1.8	1.0

TM – telemedicine visits; all – all (in person and telemedicine) visits. Diagnoses that were more frequent among telemedicine visit than all visits are highlighted in green.

© 2023 Ulyte A et al. *JAMA Network Open.*

eTable 5. Adjusted odds ratios of characteristics of SNFs in the top quartile by telemedicine use for SNF visits in 2020 and 2021

**Year**		**2020**	**2021**
**N of SNFs in top quartile**		3,618	3,318
Total N of SNFs		14,484	13,517
**Telemedicine visits in top quartile SNFs**			
	Minimum proportion	14.6%	2.3%
	Median proportion	27.3%	4.3%
**Telemedicine visits in other SNFs**			
	Median proportion	10.5%	0.4%
		**Likelihood of being in the top quartile vs other SNFs (Adjusted odds ratios and 95% CI)**	
Ownership			
	For profit	Ref	Ref
	Government	0.87 (0.72, 1.04)	0.90 (0.74, 1.09)
	Not for profit	0.98 (0.88, 1.09)	0.97 (0.87, 1.08)
Part of chain		0.93 (0.85, 1.02)	1.01 (0.92, 1.11)
Region			
	South	Ref	Ref
	Midwest	1.46 (1.32, 1.60)	1.46 (1.31, 1.62)
	Northeast	0.33 (0.28, 0.39)	0.69 (0.60, 0.80)
	West	1.03 (0.90, 1.18)	1.51 (1.31, 1.74)
Geography[a]			
	Metropolitan	ref	ref
	Micropolitan	1.62 (1.45, 1.81)	2.20 (1.96, 2.46)
	Small urban	1.33 (1.17, 1.52)	1.80 (1.58, 2.06)
	Rural	1.71 (1.47, 1.99)	2.32 (1.98, 2.71)
N of certified beds			
	≤80	ref	ref
	81-120	0.84 (0.76, 0.92)	0.84 (0.76, 0.93)
	≥121	0.68 (0.61, 0.76)	0.71 (0.63, 0.79)
Overall star rating[b]			
	1-2	ref	ref
	3-4	0.88 (0.80, 0.96)	0.91 (0.82, 1.00)
	5	0.84 (0.75, 0.95)	0.84 (0.74, 0.94)
Nurse hours per resident day			
	<1.3	ref	ref
	1.3-1.7	0.82 (0.74, 0.90)	0.91 (0.81, 1.01)
	>1.7	0.72 (0.64, 0.81)	0.76 (0.67, 0.86)
Residents on Medicaid[c]			
	≤50%	ref	ref
	51-70%	1.11 (0.99, 1.23)	1.19 (1.07, 1.32)
	≥71%	1.50 (1.34, 1.68)	1.57 (1.40, 1.77)
% resident race/ethnicity[d]			
	Black	0.87 (0.84, 0.90)	0.86 (0.83, 0.89)
	White	-	-
	Other	0.97 (0.93, 1.02)	1.06 (1.01, 1.11)

SNF – skilled nursing facility. CI – confidence interval.
a - Area type is classified based on Rural-Urban Commuting Area Codes: metropolitan (1-3), micropolitan (4-6), small urban (7-9), or rural area (10).
b - Medicare star ratings range from 1 (much below average) to 5 (much above average). This score is a composite ranking of individual SNFs that incorporates multiple measures of SNF quality, staffing, and health inspection performance.
c - The proportion of Medicaid covered residents among all Medicare residents within the SNF.
d - The mean proportion of residents of specific race/ethnicity represent the proportion of unique Medicare beneficiaries residing in the SNFs of that group. Odds ratios are provided for the variable scaled by 10 (the odds of SNF being in a high telemedicine use group associated with 10 percentage points increase in the race/ethnicity among all Medicare residents). Other race/ethnicity includes Asian, Hispanic, North American native, other, and missing.
Included are SNFs that had at least 10 SNF visits for their residents in the analyzed year. Ownership, chain, beds, and rating information is from January 2020 (CMS Nursing Homes Compare). SNFs with missing information in CMS Nursing Homes Compare are excluded (N=832 in 2020; N=1789 in 2021).

© 2023 Ulyte A et al. *JAMA Network Open.*

eTable 6. Characteristics of SNF-visit-providing clinicians in the top decile by telemedicine use in 2020-2021

	Top decile	Other clinicians	Adjusted odds ratio
N of clinicians	5,233	47,100	
N of total visits in the group	4,477,721	47,812,623	
N of total telemedicine visits in the group	1,844,057	825,663	
N of telemedicine visits (mean, SD)	352.39 (717.15)	17.53 (74.05)	
Clinician category			
Generalist medical doctor	2,728 (52.1%)	19,782 (42.0%)	ref
Specialist medical doctor	570 (10.9%)	7,508 (15.9%)	0.56 (0.50, 0.62)
Other practitioners	1,935 (37%)	19,810 (42.1%)	0.71 (0.65, 0.78)
Clinician gender: woman	2,168 (50.6%)	18,575 (48.4%)	1.29 (1.19, 1.39)
Year of graduation			
≤1995	1,440 (33.6%)	12,959 (33.8%)	ref
1996-2010	1,540 (35.9%)	11,851 (30.9%)	1.14 (1.05, 1.24)
≥2011	1,304 (30.4%)	13,519 (35.3%)	0.88 (0.79, 0.97)
Clinician geography			
Metropolitan	2,850 (66.6%)	30,746 (80.2%)	ref
Micropolitan	732 (17.1%)	3,690 (9.6%)	2.13 (1.95, 2.34)
Small town	463 (10.8%)	2,538 (6.6%)	1.84 (1.65, 2.06)
Rural	237 (5.5%)	1,367 (3.6%)	1.80 (1.56, 2.08)
N of SNF visits per clinician (mean, SD)	855.67 (1643.42)	1,015.13 (1677.6)	1.00 (0.99, 1.00)
N of SNF patients per clinician (mean, SD)	179.05 (284.7)	188.03 (256.7)	0.99 (0.98, 0.99)
N of SNFs per clinician (mean, SD)	14.79 (31.8)	10.22 (12.7)	1.03 (1.03, 1.04)

SNF – skilled nursing facility.
Clinicians in the top decile by telemedicine use had more than 18.6% of their SNF visits delivered in telemedicine. Only clinicians with at least 20 total SNF visits in 2020-2021 combined are included. Gender was missing for 9,690 (18.5%), year of graduation for 9,720 (18.6%) and geography (ZIP area information) for 9,710 (18.6%) clinicians.

© 2023 Ulyte A et al. *JAMA Network Open.*

eTable 7. Characteristics of patients who received at least one telemedicine visit during their SNF stay in 2020-2021

	**With telemedicine visit**	**No telemedicine visits**	**Adjusted odds ratio**
N	808,530 (25.5%)	2,367,988 (74.5%)	
Female sex (%)	493,322 (61.0%)	1,416,734 (59.8%)	0.96 (0.95, 0.96)
Age (%)			
<65	92,461 (11.4%)	195,819 (8.3%)	1.26 (1.24, 1.28)
65-74	196,878 (24.4%)	548,725 (23.2%)	ref
75-84	247,820 (30.7%)	766,148 (32.4%)	0.78 (0.78, 0.79)
≥85	271,371 (33.6%)	857,296 (36.2%)	0.61 (0.60, 0.62)
Race/ethnicity (%)			
Black	92,668 (11.5%)	269,303 (11.4%)	0.91 (0.90, 0.93)
White	669,920 (82.9%)	1,967,871 (83.1%)	Ref
Other	45,942 (5.7%)	130,814 (5.5%)	0.93 (0.91, 0.94)
Dual eligible in 2020 (%)	472,701 (58.5%)	893,344 (37.7%)	0.93 (0.92, 0.94)
Reason for Medicare enrollment (%)			
Old age	557,281 (68.9%)	1,792,254 (75.7%)	Ref
Disability or renal failure	251,249 (31.1%)	575,734 (24.3%)	0.96 (0.95, 0.97)
Long-term care (%)	527,568 (65.3%)	977,165 (41.3%)	0.55 (0.55, 0.55)
N of comorbidities (mean, SD)	11.3 (3.6)	10.8 (3.8)	1.07 (1.07, 1.07)
Dementia (%)	217,343 (26.9%)	463,666 (19.6%)	0.97 (0.97, 0.98)
COVID-19 during SNF stay (%)	183,235 (22.7%)	208,722 (8.8%)	1.69 (1.67, 1.70)
Limited mobility (%)	126,037 (18.4%)	346,123 (19.1%)	0.99 (0.98, 1.00)

SNF – skilled nursing facility. CI – confidence interval. SD – standard deviation.
Only residents with at least one SNF or outpatient visit during their stay were included. Model estimates are stratified by the state where SNF is located and adjusted for the length of stay at SNF with an offset (natural logarithm of total SNF stay duration in that year in days). Sociodemographic and comorbidity data was missing for 18,924 (0.6%) beneficiaries who were excluded from this table. Mobility information was missing for 585,364 (20.0%) beneficiaries. Missing mobility values were added as a separate category in the models (odds ratio not shown). Other race/ethnicity includes Asian, Hispanic, North American native, other, and missing.

© 2023 Ulyte A et al. *JAMA Network Open.*

eFigure 3. Proportion of visits delivered by telemedicine in four examined clinical care scenarios for long-term care residents in high- and low- telemedicine use SNFs in 2018-2021

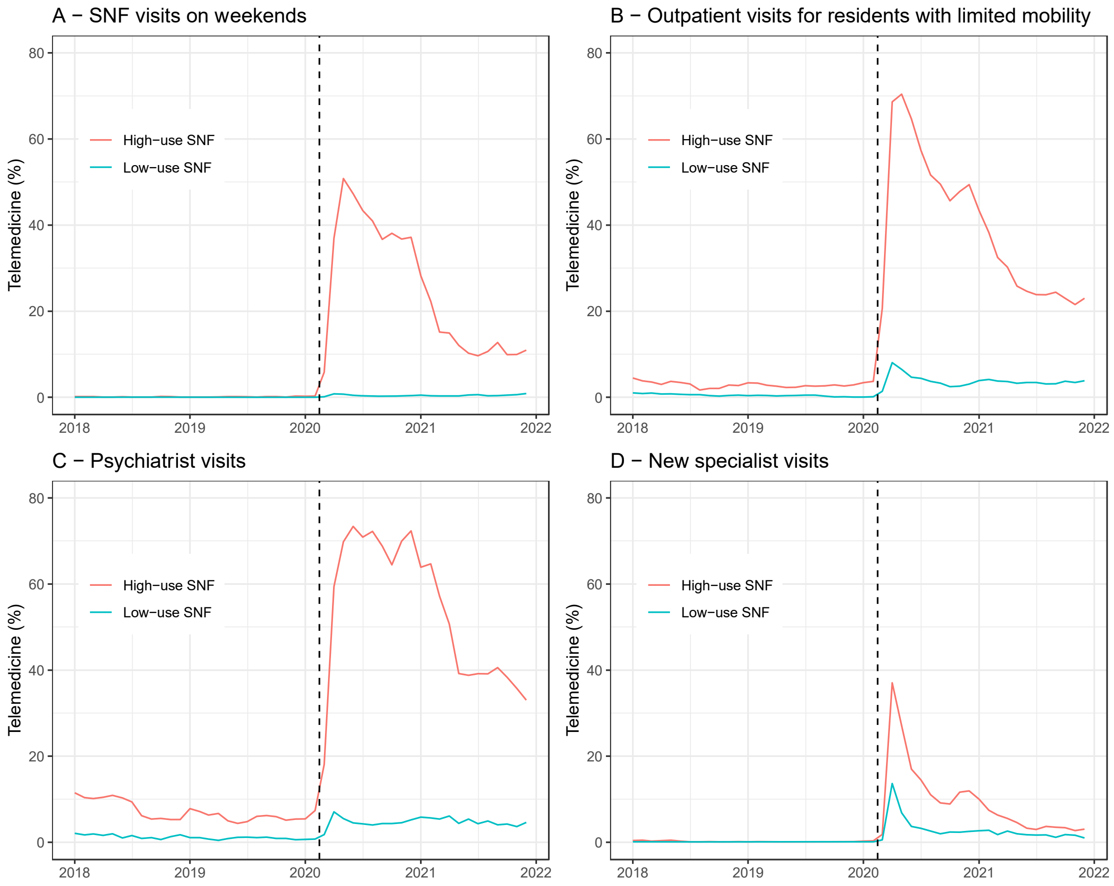

SNF – skilled nursing facility.
High-use SNFs were defined as those in the top quartile by telemedicine use in 2020 for SNF or outpatient visits (depending on the analyzed outcome), and low-use as those in the bottom quartile.

© 2023 Ulyte A et al. *JAMA Network Open.*

eAppendix 2. Testing of parallel trends for the outcomes before telemedicine expansion

To examine if the outcomes studied in the difference-in-differences analysis were evolving in parallel in 2018-2019 (before the expansion of telemedicine) in the groups of high and low telemedicine adopting SNFs, we implemented linear regression models:

$$Outcome_{iy} = \beta_0 + \beta_1 Year_y + \beta_2 SNF\_group_i + \beta_3 Year_y * SNF\_group_i + \beta_4 State_i + X_i\tau + \varepsilon_{iy}$$

$Outcome_{iy}$ is patient i's outcome in year y;
β_0 is a constant;
$Year_y$ is a linear variable for the year of the outcome;
SNF_group_i is a binary indicator of whether the patient stayed at a SNF with high telemedicine use in 2020;
$Year_y * SNF\_group_i$ is the interaction term capturing if the outcome trends in 2017-2019 in high and low telemedicine SNFs are not parallel;
$State_i$ is a fixed effect for the state where SNF is located;
$X_i\tau$ is the set of resident characteristics (indicators for sex, race, dual Medicaid eligibility, original reason for Medicare enrolment, presence of dementia, and continuous variables for the number of chronic conditions and age);
ε_{iy} is the error with SNF level clustering.

As in the main models, we weighted observations by the length of stay at SNF in days

Table below presents the estimates for the interaction term.

**Patient group –**<br>**Visits per resident-year**	β_3 **(95% confidence interval)**	**p-value**
Residents on weekends:<br>SNF visits on weekends	-0.36 (-0.64, -0.07)	0.014
Residents with limited mobility**:**<br>Outpatient visits	-0.21 (-0.33, -0.09)	0.001
All residents:<br>Outpatient psychiatrist visits	-0.04 (-0.07, -0.01)	0.004
All residents:<br>New outpatient specialist visits	0.00 (-0.01, 0.01)	0.668

The estimated interaction term is not statistically significant for new outpatient consultations with specialist physicians, which can be interpreted as linear (parallel) trends for these outcomes in 2018-2019.

It is statistically significant for outpatient visits for residents with limited mobility, SNF visits on weekends, and psychiatrist visits: in 2018-2019, high telemedicine using SNFs were increasing the number of visits slower than low telemedicine using SNFs. The differential pre-trends could mean that these groups of SNFs were potentially different in relevant ways in respect to these outcomes even before the expansion of telemedicine.

© 2023 Ulyte A et al. *JAMA Network Open.*

eAppendix 3. Difference-in-differences in visit counts in 2018-2019 vs 2020-2021 in high vs low telemedicine adopting SNFs

The results, presented in the main manuscript, were built with linear regression models, in which observations were weighted by the length of stay at SNF in days (Model 1):

$$Outcome_{iy} = \beta_0 + \beta_1 Year_y + \beta_2 SNF\_group_i + \beta_3 Period_y * SNF\_group_i + \beta_4 State_i + X_i\tau + \varepsilon_{iy}$$

$Outcome_{iy}$ is patient i's outcome in year y;
β_0 is a constant;
$Year_y$ is a fixed effect for the year of the outcome;
SNF_group_i is a binary indicator of whether the patient stayed at a SNF with high telemedicine use in 2020;
$Period_y * SNF\_group_i$ is the interaction term equal to 1 in high telemedicine use SNFs in 2020-2021 and equal to 0 in low telemedicine use SNFs and earlier years;
$State_i$ is a fixed effect for the state where SNF is located;
$X_i\tau$ is the set of resident characteristics (indicators for sex, race, dual Medicaid eligibility, original reason for Medicare enrolment, presence of dementia, and continuous variables for the number of chronic conditions and age);
ε_{iy} is the error with SNF level clustering.

The number of observations (resident-year combinations) for each model are provided below. Note that one observation reflects one resident-SNF-year pair (representing at least 60 days of SNF stay).

**Model**	**Number of resident-year observations**	
	**Total**	**High telemedicine use SNFs**
Residents on weekends: SNF visits on weekends	1,736,708	785,405
Residents with limited mobility**:** Outpatient visits	387,857	174,133
All residents: Outpatient psychiatrist visits	1,723,325	804,598
All residents: New outpatient specialist visits	1,723,325	804,598

Table 2 in the main manuscript presents estimates of the interaction term, corresponding to difference-in-differences estimate.

© 2023 Ulyte A et al. *JAMA Network Open.*

eAppendix 4. Difference-in-differences models with a separate term for 2020 and 2021

To explore if high-telemedicine use in 2020 was associated with different outcomes in year 2020 and year 2021, we implemented a model with separate terms for these years:

$$Outcome_{iy} = \beta_0 + \beta_1 Year_y + \beta_2 SNF\_group_i + \beta_3 Period_{2020} * SNF_{group_i} + \beta_4 Period_{2021} * SNF\_group_i + \beta_5 State_i + X_i\tau + \varepsilon_{iy}$$

$Outcome_{iy}$ is patient i's outcome in year y;
β_0 is a constant;
$Year_y$ is a fixed effect for the year of the outcome;
SNF_group_i is a binary indicator of whether the patient stayed at a SNF with high telemedicine use in 2020;
$Period_{2020} * SNF\_group_i$ and $Period_{2021} * SNF\_group_i$ are the interaction terms equal to 1 in high telemedicine use SNFs in 2020 and in 2021 respectively, and equal to 0 in low telemedicine use SNFs and other years;
$State_i$ is a fixed effect for the state where SNF is located;
$X_i\tau$ is the set of resident characteristics (indicators for sex, race, dual Medicaid eligibility, original reason for Medicare enrolment, presence of dementia, and continuous variables for the number of chronic conditions and age);
ε_{iy} is the error with SNF level clustering.

The estimated interaction terms are provided in the table below:

**Patient group –<br>Visits per resident-year**	**β_3 – effect in 2020**		**β_4 – effect in 2021**	
	Estimates (95% confidence interval)	p-value	Estimates (95% confidence interval)	p-value
Residents on weekends:<br>SNF visits on weekends	-0.86 (-1.27, -0.44)	0.000	-0.84 (-1.27, -0.40)	0.000
Residents with limited mobility**:**<br>Outpatient visits	0.15 (-0.04, 0.34)	0.116	0.21 (-0.01, 0.44)	0.060
All residents:<br>Outpatient psychiatrist visits	0.05 (0.02, 0.08)	0.001	0.02 (-0.03, 0.06)	0.477
All residents:<br>New outpatient specialist visits	-0.01 (-0.01, 0.00)	0.015	0.00 (0.00, 0.01)	0.227

© 2023 Ulyte A et al. *JAMA Network Open.*
