# Supplementary material for: Telemedicine Visits in US Skilled Nursing Facilities
Source: JAMA Netw Open. 2023 Aug 18;6(8):e2329895. doi: 10.1001/jamanetworkopen.2023.29895 (PMC10439478; doi:10.1001/jamanetworkopen.2023.29895)
Supplement: Supplement 2. — Data Sharing Statement [file jamanetwopen-e2329895-s002.pdf]

Data Sharing Statement

Ulyte. Telemedicine Visits in US Skilled Nursing Facilities. *JAMA Netw Open*. Published August 18, 2023. doi:10.1001/jamanetworkopen.2023.29895

Data
Data available: No

Additional Information
Explanation for why data not available: Medicare claims data are available only to specific researchers under Data Use Agreement.
